# Carbon Dioxide Embolism Resulting From Liver Laceration During Peritoneal Optical Trocar Entry

**DOI:** 10.7759/cureus.28132

**Published:** 2022-08-18

**Authors:** Andrea C Lin, Elizabeth J Olecki, Meghan L Good, Christopher Cowart, Jeffery S Scow

**Affiliations:** 1 General and Colorectal Surgery, Penn State Health Milton S. Hershey Medical Center, Hershey, USA; 2 General Surgery, Penn State College of Medicine, Hershey, USA; 3 Anesthesia, Penn State Health Milton S. Hershey Medical Center, Hershey, USA; 4 Colorectal Surgery, Penn State Health Milton S. Hershey Medical Center, Hershey, USA

**Keywords:** open and laparoscopic surgery, liver laceration, optical trocar entry, carbon dioxide embolism, air embolism

## Abstract

Venous air emboli have been reported to occur in numerous settings, including trauma, various surgical procedures, both laparoscopic and radiologically, and even idiopathically. In this case study, a liver laceration was made during a robot-assisted left colectomy and colostomy in a 69-year-old female resulting in air embolism during insufflation. A drop in end-tidal CO_2_ was noted and the patient went into immediate cardiac arrest. Adequate pressure was applied and over-suturing of the liver laceration was made with reverse Trendelenburg positioning during the administration of cardiopulmonary resuscitation (CPR) for approximately one minute. The patient completed an open hemicolectomy the following day and made a complete recovery. Preventative and intraoperative measures to prevent further recurrences of venous air emboli are discussed.

## Introduction

Venous air emboli have mostly been reported to occur secondary to iatrogenic causes [1] or traumatic injury [2]. Iatrogenic sources of venous air emboli include the use of intravenous catheters, as well as surgical procedures, in particular, laparoscopic procedures [3], with multiple instances during cholecystectomy [4-9], robot-assisted prostatectomy [10], laparoscopic appendectomy [11], hepatectomy [12], nephrectomy [13], and thyroidectomy [14]. Air emboli are also known to form during radiological procedures, including needle biopsy, hysteroscopy [15], endoscopic retrograde cholangiopancreatography [16], angiography, and during intubation sequences [17].

The occurrence of venous air embolism following iatrogenic rapid insufflation of air into venous circulation is rare, with only a few fatal events reported [18-19] and several non-fatal incidents [18-20] previously reported. Here we report an incident of iatrogenic air embolism causing cardiac arrest.

## Case presentation

**Figure 1:**
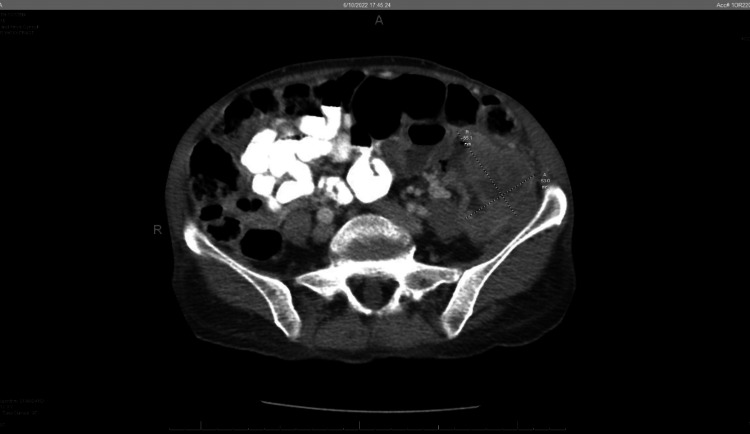
CT A/P demonstrating 65.1 mm x 53.0 mm mass in left sigmoid colon. A/P: abdomen/pelvis

A 69-year-old female was brought to the hospital by her family due to complaints of left-sided abdominal pain and weight loss. She had experienced a 10 lb weight loss and decreased appetite over the past six months. Upon admission, her weight was 35.3 kg and her BMI was 15.7 kg/m2. Physical exam was notable for mild abdominal distention and a palpable, non-tender mass in the left lower quadrant. She was anemic with a hemoglobin of 7.1 g/dl. A CT scan of the abdomen and pelvis demonstrated a large 6.5 x 5.3 cm mass in the proximal sigmoid with no evidence of disease outside the colon (Figure 1).

**Figure 2:**
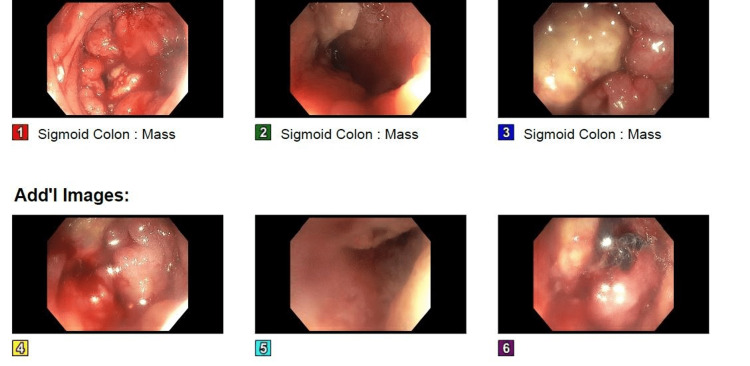
An ulcerated partially obstructing, circumferential large mass was found in the sigmoid colon at 55 cm from the anal verge. 1-3: sigmoid mass; 4-6: supplemental images.

She had never undergone a prior colonoscopy. Bowel prep was administered, and a first-time colonoscopy was performed, which revealed an obstructing mass 55 cm proximal to the anal verge (Figure 2). The colonoscope could not be advanced further.

**Figure 3:**
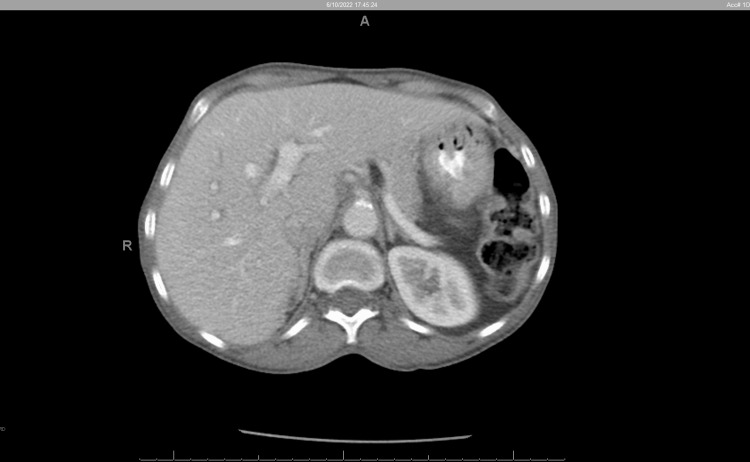
CT abdomen/pelvis obtained pre-operatively. Grossly enlarged liver showing the left lower lobe extending far beyond midline

The mass was biopsied, and pathology was positive for colon adenocarcinoma. CT scan of the chest and PET were negative for metastatic disease despite a dramatic **CEA **level of 1313 ng/ml. Preoperative CT abdomen/pelvis shows enlarged liver (Figure 3). 

The patient was scheduled for robot-assisted left colectomy and colostomy to treat the obstructing colon cancer. In the operating room, general endotracheal anesthesia was established. An optical trocar entry approach for peritoneal access in the left upper quadrant was performed, close to the edge of the rib in the midclavicular line at Palmer's point. Insufflation with carbon dioxide was initiated at "low flow" speed setting with a total pressure of 15 mmHg. The anesthesia team immediately notified the surgical team that the end-tidal CO2 decreased from 35 to 18 mmHg. Blood pressure reading began to cycle and blood was noted in the peritoneal cavity with the insertion of the laparoscope. Pneumoperitoneum was immediately released, and an air embolism was suspected.

**Figure 4:**
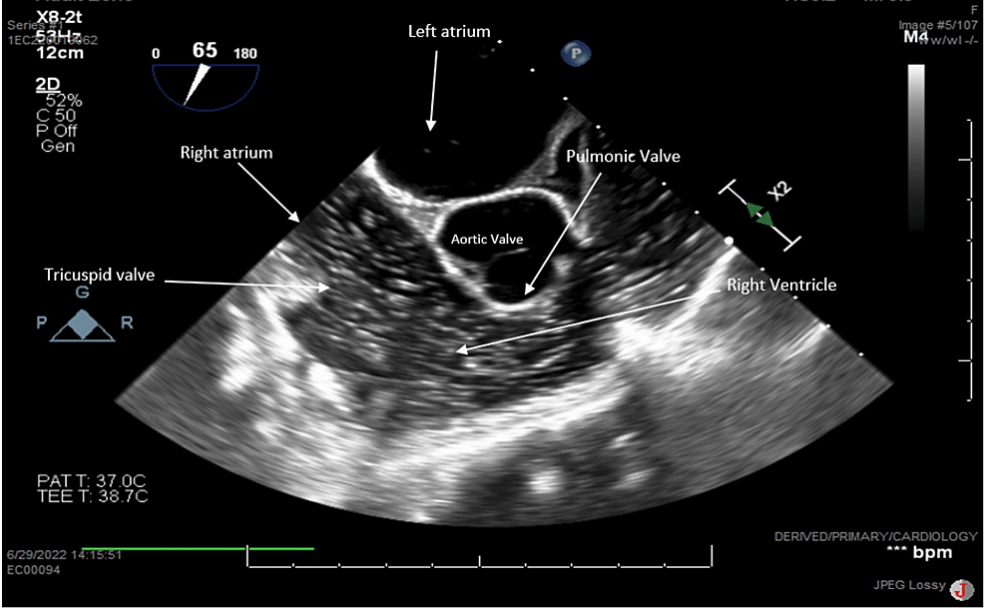
Right ventricle inflow-outflow view demonstrating significant amounts of air moving from the right atrium through the right ventricle into the main pulmonary artery. No clinically significant air seen in the left side of the heart.

Since this was most likely a CO2 embolism, the patient was treated with supportive measures with the expectation that it would resolve via diffusion through pulmonary vasculature. The patient was placed in the Trendelenburg position. The procedure was converted to an open operation via an upper midline incision and a code was initiated. Cardiopulmonary resuscitation (CPR) was immediately initiated as the patient was noted to be pulseless for approximately one minute. Compressions were performed and a total of three doses of epinephrine were administered. A 5 mm liver laceration was noted on the anterior surface of the left liver. This was controlled by applying direct pressure, electrocautery, and oversewing. A central line was placed and blood and air were aspirated. A transesophageal echocardiogram revealed a large amount of air visible in the right heart (Figure 4).

There was a decreased function of the right ventricle. The left ventricle appeared to be functionally normal with no visible patent foramen ovale or aortic dissection. Air was also visualized in the inferior vena cava and hepatic vein. Less than 50 ml of blood loss was reported. Temporary abdominal closure was performed and the operation was aborted.

The patient was transported to the intensive care unit (ICU) where she remained intubated and mechanically ventilated. Post-operatively, a normal biventricular function was reported, and vasopressor support was weaned. Overnight, the patient had a complete neurological assessment and recovery. She was deemed stable for open hemicolectomy with a colostomy the following day. The operation was successfully performed, and she was extubated in the ICU on the day of surgery. Her postoperative convalescence was notable for ileus treated conservatively. She was discharged home 11 days after the colectomy.

## Discussion

In our case, a superficial 5 mm liver laceration during optical peritoneal entry allowed carbon dioxide to enter venous circulation during insufflation despite the use of low speed and low pressure of 15 mmHg. This was confirmed via transesophageal echocardiogram which visualized air in the portal venous system with no other associated injury to vessels in the abdominal cavity. If this CO2 embolism had been due to the peritoneal entrainment of CO2, we would not have seen gas in the portal system. Air emboli are a rare occurrence but can be deadly [1]. For these particular emboli, air accumulation within the right ventricle causes heart contractions to become inadequate. This results in obstructive shock, acute right ventricular failure, or pulselessness which occurred in our patient [9].

Typically, emergent treatment of an air embolism entails repositioning the patient via Durant’s maneuver, which consists of left-lateral decubitus and head-down positioning [8]. This allows for less air to enter the right ventricular outflow tract. Additionally, in patients with a patent foramen ovale, a paradoxical embolism can occur, as the air can pass into the systemic circulatory system, which had been assessed for in this patient.

Aggressive fluid resuscitation to prevent shock along with the administration of vasopressors and/or inotropes are recommended supportive therapies. Another controversial recommendation is the administration of 100% O2 or hyperbaric oxygen therapy (HOT) with the goal of dissolving trapped nitrogen [16], with some sources explaining that delayed administration of HOT decreases patient mortality [17]. It is possible we could have initiated treatment with hyperbaric oxygen. However, our facility does not have this capability and this embolism was due to insufflation with carbon dioxide and not nitrogen-containing air.

## Conclusions

Ultimately, although air embolism in this setting is incredibly rare, the risk of fatality is quite high. Accidental liver laceration upon laparoscopic entry can lead to air embolism. Prevention of liver injury during optical trocar entry may be improved with preoperative imaging to visualize and avoid the lower border of the liver. Intraoperatively, test insufflation with several mL of carbon dioxide and slow insufflation with low pressures may reduce the incidence of large air emboli. In the case that air embolism does occur, techniques to help stabilize the patient following onset can improve patient outcomes and decrease mortality. Intraoperative measures and supportive therapies include transitioning to Trendelenburg or Durant’s position, administration of vasopressors and/or inotropes, and in some cases, hyperbaric oxygen therapy.
